# Selective Inhibition of Human Monoamine Oxidase B by 5-hydroxy-2-methyl-chroman-4-one Isolated from an Endogenous Lichen Fungus *Daldinia fissa*

**DOI:** 10.3390/jof7020084

**Published:** 2021-01-26

**Authors:** Geum-Seok Jeong, Myung-Gyun Kang, Sang-Ah Han, Ji-In Noh, Jong-Eun Park, Sang-Jip Nam, Daeui Park, Sung-Tae Yee, Hoon Kim

**Affiliations:** 1 Department of Pharmacy, and Research Institute of Life Pharmaceutical Sciences, Sunchon National University, Suncheon 57922, Korea; fever41@naver.com (G.-S.J.); nji8009@naver.com (J.-I.N.); park140201@naver.com (J.-E.P.); sungtae@scnu.ac.kr (S.-T.Y.); 2 Department of Predictive Toxicology, Korea Institute of Toxicology, Daejeon 34114, Korea; myung-gyun.kang@kitox.re.kr (M.-G.K.); daeui.park@kitox.re.kr (D.P.); 3 College of Pharmacy, Ewha Womans University, Seoul 03760, Korea; tkddk9559@naver.com; 4 Department of Chemistry and Nanoscience, Ewha Womans University, Seoul 03760, Korea; sjnam@ewha.ac.kr

**Keywords:** endogenous lichen fungus, *Daldinia fissa*, 5-hydroxy-2-methyl-chroman-4-one, selective monoamine oxidase B inhibitor, blood–brain barrier permeability, docking simulation

## Abstract

Inhibitory activities against monoamine oxidases (MAOs) and cholinesterases (ChEs) and antioxidant activity were evaluated for 195 extracts from Ukraine-derived endogenous lichen fungi (ELF). Among them, an ELF13 (identified as *Daldinia fissa*) extract showed the highest inhibitory activity against MAO-B, and 5-hydroxy-2-methyl-chroman-4-one (HMC) was isolated as a ~ 4-fold selective inhibitor of MAO-B (IC_50_ = 3.23 µM) compared to MAO-A (IC_50_ = 13.97 µM). HMC is a reversible competitive inhibitor with a K_i_ value of 0.896 µM. No cytotoxicity was observed in normal and cancer cells at 50 µM of HMC. HMC showed blood–brain barrier permeability and high gastrointestinal absorption in silico pharmacokinetics. The docking simulation results showed that the binding affinity of HMC for MAO-B (−7.3 kcal/mol) was higher than that of MAO-A (−6.1 kcal/mol) and that HMC formed a hydrogen bond interaction with Cys172 of MAO-B (distance: 3.656 Å), whereas no hydrogen bonding was predicted with MAO-A. These results suggest that HMC can be considered a candidate for the treatment of neurodegenerative diseases, such as Alzheimer’s disease and Parkinson’s disease.

## 1. Introduction

Lichens are complex life forms in which algae and fungi maintain a symbiotic relationship. They not only live in extreme environments, such as tundra, desert, and volcanic areas in the polar regions, but also grow in various places, such as rainforests and temperate regions [1]. The primary and secondary metabolites of lichens have excellent pharmacological effects, such as anti-cancer, anti-inflammatory, osteoporosis relief, and anti-biotic, and thus research to identify source materials for new drug development has been underway [2,3]. The species of *Thamnolia vermicularis* (Sw.) Schaer. exist in a variety of environments, such as gravel, frost boils tundra, and mossy bushes [4]. The polysaccharide extract of this lichen has shown various biological activities such as antitumor and immunomodulatory activities [5].

On the other hand, Alzheimer’s disease (AD) is the most common degenerative brain disease that causes dementia [6]. It develops very slowly and is characterized by a gradual progression. In the early stages, it mainly causes problems with memory for recent events and is accompanied by other cognitive abnormalities, such as speech and judgment, eventually losing all daily life functions [7]. In AD, an excessive accumulation of β-amyloid is known as a key mechanism [8], and the activities of monoamine oxidase (MAO) and cholinesterases (ChEs) induce AD [9]. The number of patients with AD is increasing each year worldwide; therefore, it is important to identify an effective way to treat AD. In addition, depression is a phenomenon in which depressed mood, interest, and enjoyment decrease, resulting in discouragement, hopelessness, and sometimes suicidal thoughts [10]. It is known that the reduction of neurotransmitter monoamines in neuronal synapses, such as norepinephrine and serotonin, is the main cause of depression, MAO breaks down monoamines and causes depression [11].

MAO is an enzyme that catalyzes the oxidative deamination of neurotransmitter monoamines and has two isoforms, A and B [12]. MAO-A is associated with neuropsychiatric disorders, such as depression, and MAO-B is related to neurodegenerative diseases, such as AD and Parkinson’s disease (PD) [13]. ChEs are classified into acetylcholinesterase (AChE) and butyrylcholinesterase (BChE). AChE breaks down acetylcholine (ACh) into acetate and choline [14]. ACh is most widely distributed in the cerebral cortex and is known to be a representative neurotransmitter. AD patients appear to be deficient in the amount of ACh in the brain. BChE breaks down butyrylcholine, and the level of BChE is high in the cerebrum of AD patients [15,16]. In addition, β-site amyloid precursor protein cleaving enzyme 1 (β-secretase, BACE-1) affects the progression of AD [17,18].

In this study, the inhibitory activities against MAO-A, MAO-B, AChE, and BChE for a library of 195 extracts of endogenous lichen fungi (ELF) were evaluated as well as antioxidant activity, and 5-hydroxy-2-methyl-chroman-4-one (HMC) was isolated from an extract of ELF13, identified as *Daldinia fissa*, as a strong MAO-B inhibitor. In addition, an inhibitory activity for BACE-1, kinetic studies, cytotoxicity tests, in silico pharmacokinetics, and docking simulations were carried out for the compound HMC.

## 2. Materials and Methods

### 2.1. Extracts of Endogenous Lichen Fungi and Evaluations of Inhibitory Activities

A library of 195 extracts with ethyl acetate (EA) or butanol (BuOH) of endogenous lichen fungi (ELF) derived from Ukraine was obtained from Korea Lichen Research Institute (KoLRI) in Sunchon National University, Republic of Korea. The extracts were tested for their inhibitory activities against MAO-A, MAO-B, AChE, and BChE, and their antioxidant activity. All chemicals and enzymes were purchased from Sigma-Aldrich (St Louis, MO, USA), unless otherwise specified.

### 2.2. Monoamine Oxidase (MAO) Activity Assay

Human recombinant MAO-A and MAO-B enzymes were used, and the activities were measured in a final volume of 500 µL of 100 mM sodium phosphate buffer of pH 7.2, and 0.06 mM kynuramine (MAO-A), or 0.3 mM benzylamine (MAO-B). MAO-A and MAO-B activities were assayed at 316 nm and 250 nm, respectively, for 30 min in the kinetic mode [19,20].

### 2.3. Cholinesterase Activity Assay

For cholinesterase activity assay, electric eel AChE and horse serum BChE were used. Activities were measured according to the Ellman method [21], with slight modifications [22,23], in 50 mM sodium phosphate buffer (pH 7.5). After adding cholinesterase enzymes and the inhibitor, pre-incubation was applied for 15 min. After adding 0.5 mM 5,5-dithiobis (2-nitrobenzoic acid) (DTNB) 0.5 mM for color development, 0.5 mM acetyl thiocholine iodide (ATCI) or 0.5 mM butyrylthiocholine iodide (BTCI) was used as a substrate. The activities were measured in a final volume of 500 µL in the kinetic mode for 15 min at a wavelength of 412 nm.

### 2.4. β-Site Amyloid Precursor Protein Cleaving Enzyme 1 (BACE-1) Activity Assay

BACE-1 assay was carried out using the BACE-1 activity detection kit and a fluorescence spectrometer (FS-2, Scinco, Seoul, Korea) by an excitation at 320 nm and an emission at 405 nm, after the reaction for 2 h at 37 °C [24].

### 2.5. Antioxidant Activity Assay

The antioxidant activity was measured at 100 µg/mL of the extracts using 0.1 mM 2,2-diphenyl-1-picrylhydrazyl (DPPH). After 15 min of pre-incubation, absorbance was measured at 517 nm [25].

### 2.6. Culture and Extraction of the Selected Endogenous Lichen Fungi

The selected ELF were cultured in potato dextrose broth (PDB) agar medium. The cultured fungi were inoculated into 200 mL of PDB liquid medium by adding 5 × 5 mm (3–4 pieces) and incubated at 20 °C and 150 rpm for 2 weeks. The culture solution was suspended with a homogenizer (HG-15A, Daihan Sci. Co., Wonju, Korea) and extracted with the same volume of EA for 2 h at 20 °C and 150 rpm. The supernatant was filtered through a Whatman No. 1 filter, then concentrated, and recovered through a vacuum rotary concentrator.

### 2.7. Thin-Layer Chromatography

Thin-layer chromatography (TLC) was performed to separate constituents in the extract using Prep TLC plates (PTLC Silica gel 60 F_254_, 0.5 mm, Merck, Darmstadt, Germany). The extract was developed, and the spots on the chromatography were recovered under 254 nm through an activity-guided method. The first solvent was ethyl acetate and toluene in a ratio of 1:9 (*v*/*v*), and the second solvent was chloroform and toluene in a ratio of 1:9 (*v*/*v*). The concentration of the sample used was 100 mg/mL, and a maximum volume of 500 µL was loaded onto the PTLC plate.

### 2.8. Structure Analysis through Nuclear Magnetic Resonance (NMR), Liquid Chromatography–Mass Spectrometry (LC-MS)

Nuclear magnetic resonance (NMR) spectra were recorded with an NMR spectrometer (Varian Medical Systems, Inc., VA, USA) at 400 MHz for ^1^H and 100 MHz for ^13^C in MeOD using a solvent signal as an internal reference (δ_H_ 4.870/δ_C_ 49.150). Optical rotations were acquired using a polarimeter (Optronic P-8000, Kruss, Hamburg, Germany) with a 5 cm cell.

### 2.9. Inhibitory Activities of the Compound and Enzyme Kinetics

The inhibitory activities of the isolated compounds were analyzed at a concentration of 2 µg/mL. After measuring the IC_50_ value of the compound, the K_i_ value was determined using Lineweaver-Burk plots at ~1/2 × IC_50_, IC_50_, and 2 × IC_50_ concentrations, and a secondary plot obtained by the slope versus the inhibitor concentrations [23].

### 2.10. Inhibitor Reversibility Analysis

The reversibility analysis of the compound was performed by a recovery experiment through dialysis using 100 mM sodium phosphate (pH 7.2) buffer at 2 × IC_50_ [26]. For the reversibility analysis of MAO-B, the reversible inhibitor lazabemide and the irreversible inhibitor pargyline were used as references.

### 2.11. Cytotoxicity Analysis of the Compound

Cell viability was determined according to the cell counting kit (CCK)-8 assay method [27], using MDCK (Madin–Darby canine kidney) and HL-60 (human acute promyelocytic leukemia) cells, as normal and cancer cells, respectively. Briefly, MDCK and HL-60 cells were resuspended in Dulbecco’s modified eagle medium (DMEM) and Roswell Park Memorial Institute (RPMI)-1640, respectively, at 1 × 10^5^ and 3 × 10^5^ cells/mL, respectively. The cell suspension (100 µL) was added to each well of the 96-well plate and was incubated for 24 h at 5% CO_2_ and 37 °C. After incubation, 100 µL of the medium supplemented with 1, 3, 10, 30, and 50 µM of the compound was added to each well and incubated again. After 24 h, 100 µL of the solution was removed from each well, and CCK-8 (10 µL/well) was dispensed. After 2~4 h, the absorbance was detected at 450 nm with a microplate reader (Versamax, Molecular Devices, CA, USA).

### 2.12. Docking Simulations and Molecular Dynamics of the Compounds with Monoamine Oxidase-A (MAO-A) and MAO-B

To simulate the dockings of (*S*)- or (*R*)-HMC to MAO-A or MAO-B, AutoDock Vina plugin in UCSF Chimera 1.14 (build 42094) was used, which has an automated docking facility [28,29]. To define the docking sites of hMAO-A and hMAO-B, the predefined active sites in MAO-A/7-methoxy-1-methyl-9H-beta-carboline (HRM) complex (PDB ID: 2Z5X) [30] and MAO-B/pioglitazone complex (P1B) (PDB ID: 4A79) [31] were used. To prepare target proteins, all molecules including water except the flavin-adenine dinucleotide (FAD) were removed from the target structures, and then hydrogens and charges were added. For the docking simulation, the 2D structures of the compounds were created and converted into 3D structures, and energy minimization was conducted with the ChemOffice 2002 (ChembridgeSoft). Based on the results of the docking simulation, we checked for possible hydrogen bonding using the bonding relaxation constraints of 0.4 Å and 20.0° using Chimera [32]. The amino acids within 4Å from the docked poses were depicted as key residues, and the FAD was also displayed together to show the distances from the docked compounds. We performed molecular dynamics of HMC enantiomers complexed with MAO-A and MAO-B using NAMD 2.21 [33] and VMD 1.9.4 [34] software, and applied the CHARMM 36 parameters to the analysis at CHARMM-GUI website (http://www.charmm-gui.org/) [35], with 10,000 steps as the initial minimum at 310K. The data were examined using the root mean square deviation (RMSD) by time and the structure variation was calculated by RMSD values of protein-ligand complexes from 0 to 1000 ps.

### 2.13. Pharmacokinetic Analysis of the Compound Using the In Silico Method

The pharmacokinetic and physicochemical analysis of the compound was performed using the web tool of SwissADME (http://www.swissadme.ch/), and gastrointestinal absorption, blood-brain barrier permeability, P-glycoprotein substrate, and cytochrome P450 inhibitory activities were analyzed [36].

## 3. Results

### 3.1. Inhibitory Activities against the Enzymes and Antioxidant Activities of the Extracts

Inhibitory activities against MAO-A, MAO-B, AChE, and BChE were primarily tested for 195 species of ELF extracts from Ukraine at 20 (MAO) or 50 µg/mL (ChE). The samples were screened based on the residual activities (Figures S1–S4 in Supplementary Materials). The cutoff values were 30% for MAO-B and 50% for MAO-A, AChE, and BChE. Thus, two samples for MAO-A, five for MAO-B, two for AChE, and one for BChE were selected (Table 1). ELF13 showed the lowest residual activity (19.7%) for MAO-B; however, it exhibited no significant inhibitory activities for other enzymes. Therefore, ELF13 was selected for further study and subjected to the cultivation, extraction, and isolation of the MAO-B inhibitor. ELF13 was identified as a fungus *Daldinia fissa* forming a symbiotic relationship with the lichen *Thamnolia vermicularis* (Sw.) Schaer. On the other hand, antioxidant activity of 195 extracts of ELF was primarily measured at 100 µg/mL (Figure S5), and three extracts were selected based on the result of the DPPH antioxidant activity analysis (Table 2). ELF87 showed the highest inhibition (84.8%), followed by ELF8 (58.5%) at 100 µg/mL.

### 3.2. Isolation of Compounds from Endogenous Lichen Fungi 13 (ELF13) Using Prep Thin-Layer Chromatography (TLC)

**Figure 1:**
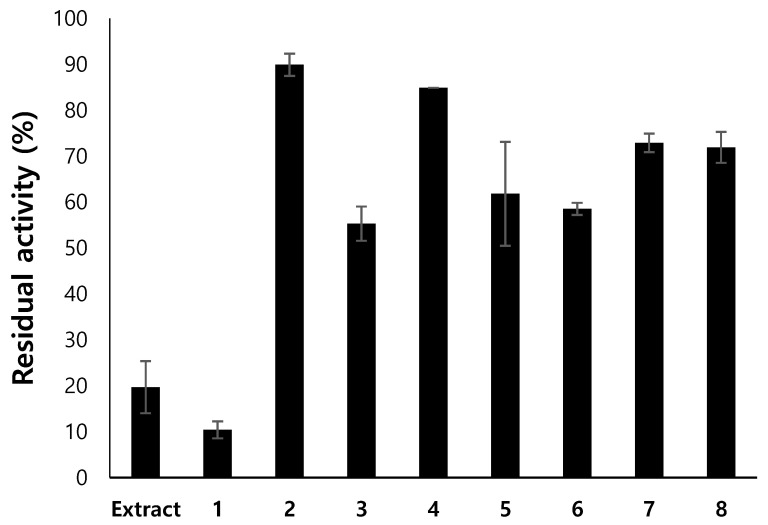
Residual activities of the ELF13 extract and eight fractions from primary PTLC. The compound was separated with the first solvent (ethyl acetate:toluene = 1:9, *v*/*v*). The activity of the compound was measured at 20 µg/mL.

A total 6 L of the culture (200 mL × 30 flasks) of ELF13 was extracted and concentrated to isolate compounds. Eight spots appeared on the PTLC plate with the primary solvent and compounds in the spots were recovered. The inhibitory activities against MAO-B by the compounds were confirmed through the activity-guided method. Among the recovered eight fractions, fraction 1 showed the lowest residual activity (10.4%), and other fractions showed higher residual activities of >50% (Figure 1). In the additional PTLC with the secondary solvent, two spots, **C1** and **C2**, were identified and recovered to be 1 mg and 17 mg (≥96.8%, purity checked by high-performance liquid chromatography (HPLC)), respectively, from 600 mg of fungal extract. For the inhibitory activity analysis, **C2** showed 13.2% and 55.8% of residual activity for MAO-B and MAO-A, respectively, at 2 µg/mL; however, no significant inhibitory activity was observed for AChE, BChE, or BACE-1 (Table 3). C1 showed very weak inhibitory activities for the enzymes. **C1** and **C2** showed very weak antioxidant activity (Table 3). Because compound **C1** showed no significant inhibitory activities and had an amount limitation, only compound **C2** was further studied.

### 3.3. Molecular Structure Analysis of C2

**Figure 2:**
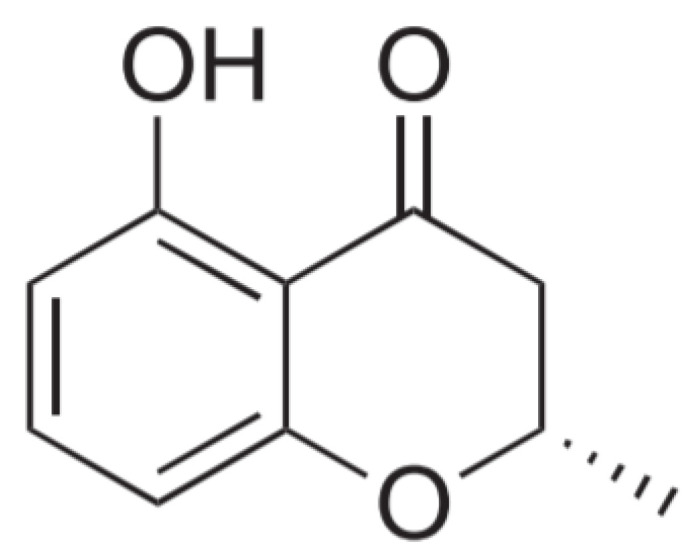
Molecular structure of **C2**; 5-hydroxy-2-methyl-chroman-4-one (HMC).

The ^1^H NMR spectrum of **C2** revealed three aromatic protons [H-6 (δ_H_ 6.43), H-7 (δ_H_ 7.38), H-8 (δ_H_ 6.46)], one methylene proton [H-3 (δ_H_ 2.73)], one methine proton [H-2 (δ_H_ 4.55)], and one methyl doublet proton [H-2 (δ_H_ 1.49)] (Figure S6). The ^13^C NMR and HMBC spectroscopic data displayed one carbonyl carbon [C-4 (δ_C_ 199.6)], one oxygenated carbon [C-2 (δ_C_ 75.3)], one methylene carbon [C-3 (δ_C_ 42.8)], three quaternary carbons [C-10 (δ_C_ 109.0), C-5 (δ_C_ 163.3), C-9 (δ_C_ 163.2)], three aromatic carbons [C-6 (δ_C_ 108.4), C-7 (δ_C_ 139.0), C-8 (δ_C_ 109.7)], and one methyl carbon [C-2 (δ_C_ 21.0)] (Figures S7 and S8). The LR-ESI-MS data of **C2** showed the peak of *m*/*z* 179.2 [M+H]^+^ (Figure S9). Compound **C2** was identified as 5-hydroxy-2-methyl-chroman-4-one (HMC) (also known as 2,3-dihydro-5-hydroxy-2-methyl-4H-1-benzopyran-4-one) (Figure 2), based on the comparison of the NMR spectroscopic data with those previously reported one [37]. The optical rotation of **C2** ([α]_D_^22^ = −40, *c* 0.0013, methylene chloride) suggested that **C2** exists as an excess of the (*S*) enantiomer of 5-hydroxy-2-methyl-chroman-4-one [38].

### 3.4. Inhibitory Activities of HMC (5-hydroxy-2-methyl-chroman-4-one) against the Enzymes

**Figure 3:**
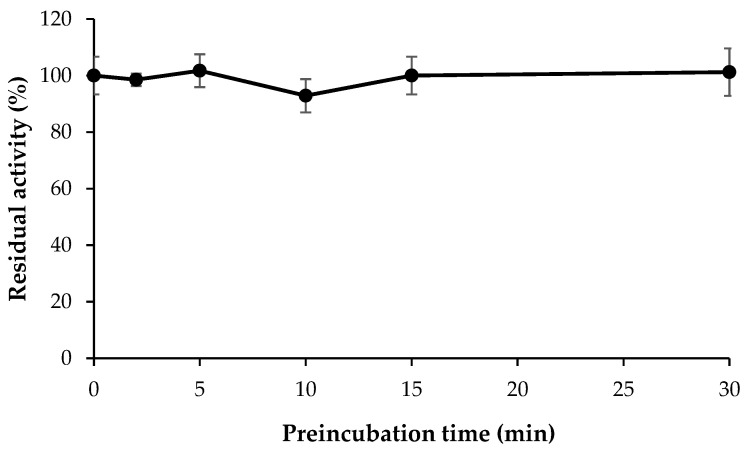
Time-dependency of HMC and MAO-B. The activities were measured after preincubation to the designated times, and were expressed as residual activities compared with control.

In time-dependency of HMC and MAO-B, the activities were measured after preincubation up to 30 min. The residual activities were not changed significantly, indicating a time-independent manner (Figure 3). For the inhibitory activities of HMC against the enzymes, IC_50_ values for MAO-A and MAO-B were 13.97 and 3.23 µM, respectively (Table 4). The values for AChE and BChE were >40 µM.

### 3.5. Inhibition Patterns of HMC

**Figure 4:**
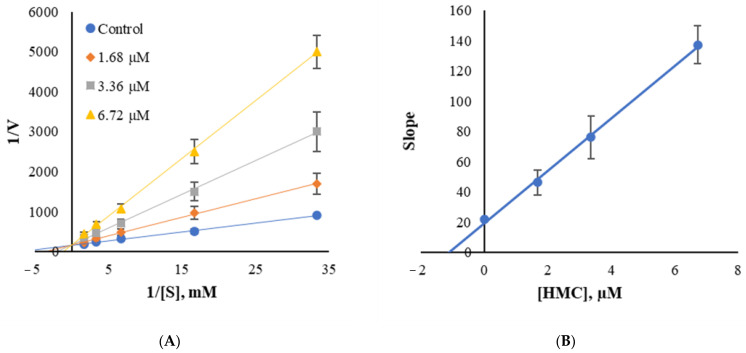
Lineweaver–Burk plots (**A**) and the secondary plot (**B**) of HMC for MAO-B.

The inhibition plot for MAO-B indicated that it is a competitive inhibitor encountered on the y-axis in Lineweaver–Burk plots (Figure 4A). The secondary plot of the slope of the Lineweaver–Burk plots as a function of inhibitor concentration showed a K_i_ value of 0.896 µM for MAO-B (Figure 4B).

### 3.6. Inhibitor Reversibility of HMC

**Figure 5:**
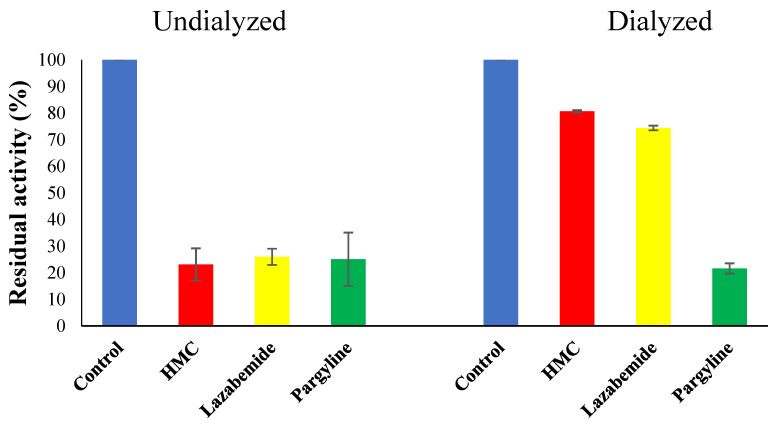
Inhibition of HMC of MAO-B and recovery activity by dialysis. Concentrations of ~2× IC_50_ were used.

In the reversibility test of HMC, lazabemide and pargyline were used as references as a reversible and irreversible inhibitor for MAO-B, respectively. In the dialysis experiments, the inhibition of MAO-B by HMC was recovered from 23.1% to 80.6%, similar to lazabemide, from 26.0% to 74.4%, but not to pargyline, which was not recovered at all. This result confirms that HMC is a reversible inhibitor (Figure 5).

### 3.7. Cytotoxicity of HMC

**Figure 6:**
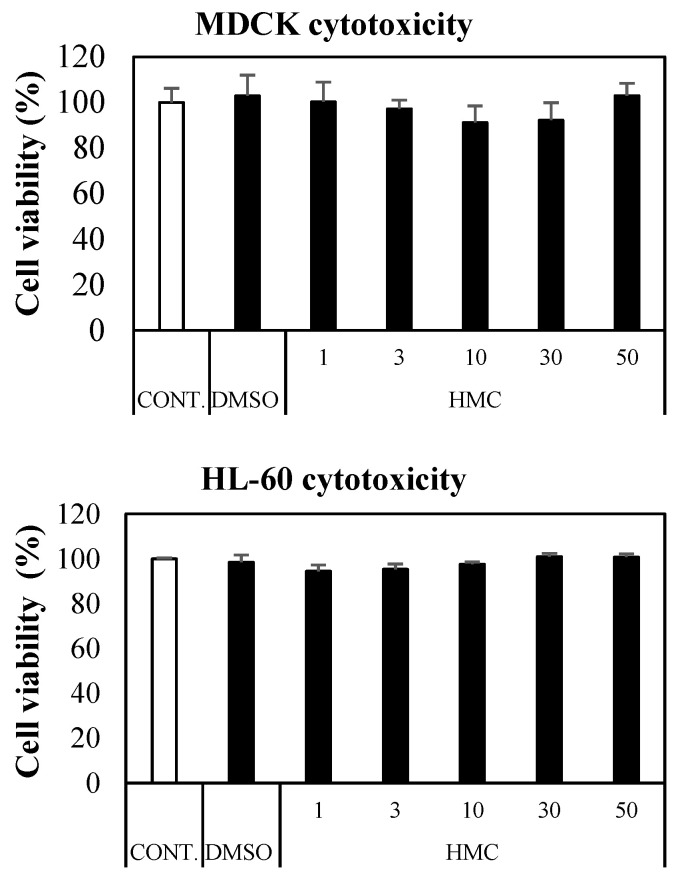
Cytotoxicity of HMC for MDCK (a normal cell line) and HL-60 (a Cancer cell line). The cells were treated with HMC (1, 3, 10, 30 and 50 µM) for 24 h. The culture supernatant was removed, and cell counting kit-8 was added. All data are expressed as mean ± standard deviation (SD) of triplicate independent experiments.

For the cytotoxicity tests, cell viabilities were not decreased at all for MDCK (normal cell line, for 3 h culture) or HL-60 (cancer cell line, for 4 h culture) cells with the treatment of 50 µM HMC (Figure 6).

### 3.8. Molecular Docking Simulation and Molecular Dynamics

**Figure 7:**
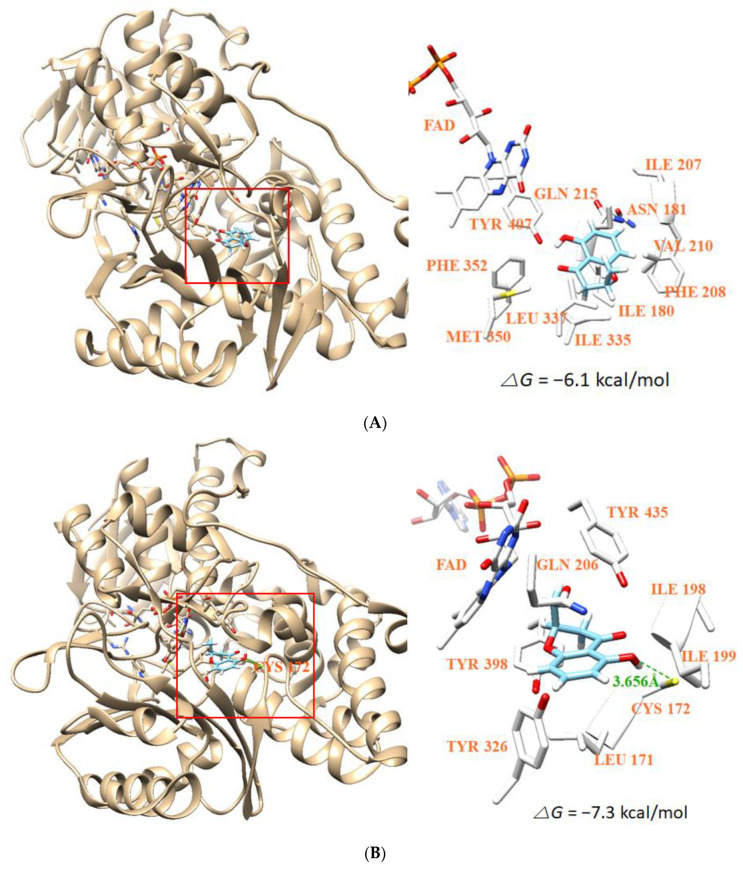

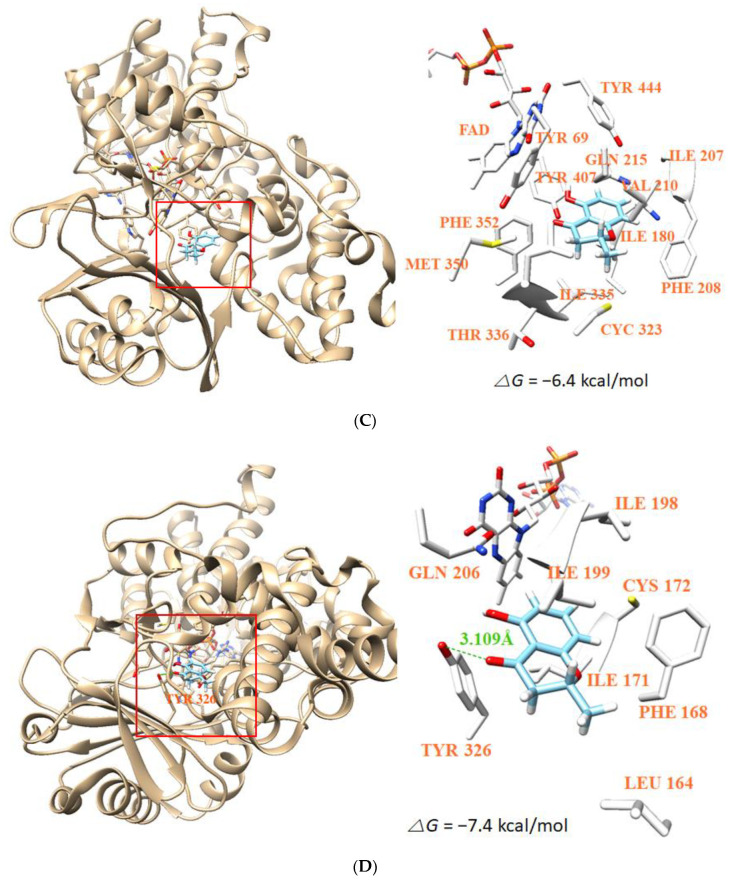

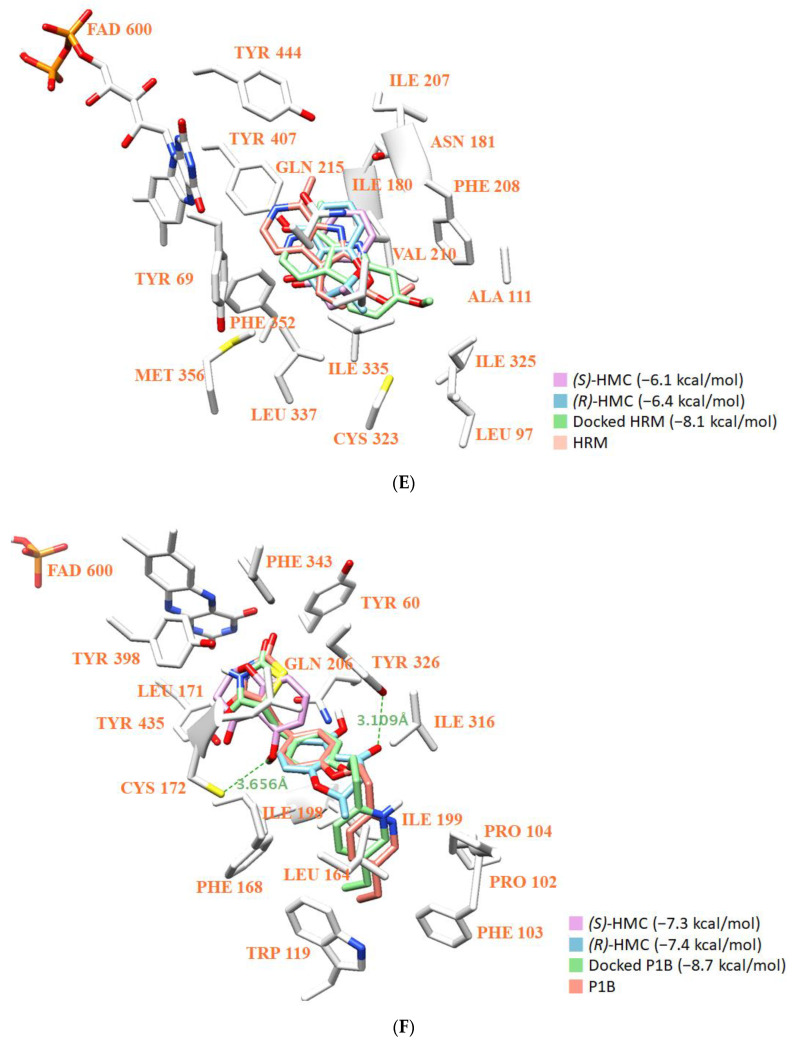
Docking simulations of (*S*)- and (*R*)-HMC to MAO-A (**A**,**C**, respectively) and MAO-B (**B**,**D**, respectively) and positioning of (*S*)-, (*R*)-HMC, docked, and co-crystallized ligands to MAO-A (**E**) and to MAO-B (**F**). MAO-A (PDB: 2Z5X) and MAO-B (PDB: 4A79) were subjected to docking analyses.

The docking simulations showed that (*S*)-HMC located well at the binding site of HRM complexed with MAO-A and at the binding site of P1B complexed with MAO-B. The AutoDock Vina showed that the binding affinity of the compound for MAO-B (−7.3 kcal/mol) was higher than that of MAO-A (−6.1 kcal/mol), and that the compound could interact with MAO-B by a hydrogen-bond with the Cys172 residue at a distance of 3.656 Å, whereas no hydrogen bond interaction was predicted for MAO-A (Figure 7A,B). When (*R*)-enantiomer was analyzed, the binding affinities for MAO-B (−7.4 kcal/mol) and MAO-A (−6.4 kcal/mol) were similar or comparable to (*S*)-enantiomer (Figure 7C,D). To validate these results, the docking simulation with co-crystallized ligands, HRM (K_i_ = 5 or 17 nM) and P1B (K_i_ = 500 nM) were used for MAO-A and MAO-B, respectively, and their binding scores were calculated to be −8.1 kcal/mol and −8.7 kcal/mol, respectively (Figure 7E,F). Interestingly, *S*-enantiomer bound to a deeper position at the active site of MAO-B than *R*-enantiomer, which was positioned at a centered space, with a reverse conformation of the chiral carbon atom (Figure 7F).

In molecular dynamics, for both MAO-A and MAO-B complexes, the RMSD values increased and reached a stable state after 125 ps. The RMSD values in complexes with MAO-B and HMC enantiomers were lower than those in MAO-A (Figures S10 and S11). The average RMSD values of (*S*)-HMC for MAO-B and MAO-A were estimated to be 0.971 ± 0.121 and 1.061± 0.172 Å, respectively, and those of (*R*)-HMC for MAO-B and MAO-A were 0.860 ± 0.097 and 1.017 ± 0.181 Å, respectively.

### 3.9. In Silico Pharmacokinetics of HMC

The pharmacokinetic analysis of HMC using SwissADME’s web tool showed that it has high gastrointestinal absorption and can cross the blood-brain barrier, and does not inhibit cytochrome P450 (Table 5). For Lipinski parameters of HMC, it was predicted that there were no violations of Lipinski’s rule of five (Table 6). These properties can provide advantages for central nervous system (CNS) drugs.

## 4. Discussion

In this study, the inhibitory activities of 195 ELF extracts from Ukraine-derived lichens against MAO-A, MAO-B, AChE, and BChE were evaluated as well as their antioxidant activities. Among them, several effective extracts were selected: two extracts for MAO-A, five for MAO-B, two for AChE, one for BChE inhibitory activities, and three for antioxidant activity. Among them, ELF13 extract showed the highest inhibitory activity against MAO-B. ELF13 was an endogenous fungus *Daldinia fissa* forming a symbiotic relationship with the lichen *Thamnolia vermicularis* (Sw.) Schaer.

Extracts and compounds of lichens were shown to have effective inhibitory activities against various enzymes, including MAO and AChE in a recent review [39]. However, little information is available: Solorinic acid of anthraquinones inhibited MAO enzyme with IC_50_ 14.3 µM [40]. A synthetic derivative 4-acylresorcinol displayed potent inhibitory activity with IC_50_ value 4.27 µM, among lichen compounds and their synthetic analogues [41]. The inhibitory activities were determined using liver or brain MAO and the IC_50_ values of the compounds were higher than that of HMC, which showed strong and ~4-fold selective MAO-B inhibitory activity (IC_50_ = 3.23 µM).

HMC is known to have several biological activities, such as antifungal and phytotoxicity [42], and an inhibition of *Saccharomyces cerevisiae* growth and the formation of soybean callus [37]. Mellein, an isochromane, is known to inhibit monoamine oxidase [43], HCV protease [44], hepatitis C virus protease [45], and human DNA polymerase lambda [46]. The potency of HMC for MAO-B is much higher than that of mellein (IC_50_ = 8.93 µg/mL, 50.16 µM)) [43].

Lazabemide (IC_50_ = 0.063 µM) is a reversible inhibitor of MAO-B and is used in the treatment of PD and AD. The potency of HMC is lower that of lazabemide; however, it can be served as a lead compound or a scaffold for the development of promising derivatives through the large-scale cultivation of fungi as a natural compound.

The docking simulations revealed that HMC interacted with the Cys172 of MAO-B to form a hydrogen bond, while no hydrogen bond interaction with MAO-A was predicted. Therefore, hydrogen bond interaction might play an important role in the strong binding and inhibitory activity of HMC against MAO-B. Although the positions and orientations of two (*S*)- and (*R*)-enantiomers at the MAO-B active site were different, few differences in binding affinities were predicted. Collectively, binding affinity of HMC to MAO-B is greater than that of MAO-A, and the affinities of *(S)*-enantiomer to the enzymes are comparable to those of *(R)*-enantiomer. Molecular dynamics also supported the experimental data and the docking simulations well. In addition, HMC crosses the blood–brain barrier and shows no violations of Lipinski’s rule of five, and no intracellular toxicity, indicating pharmacological potential. This indicates that HMC can be considered a candidate for the treatment of neurodegenerative diseases.

## 5. Conclusions

HMC, isolated from an ELF extract, showed a strong inhibitory activity against MAO-B (IC_50_ = 3.23 µM) with a moderate selectivity over MAO-A (IC_50_ = 13.97 µM), and is a reversible competitive inhibitor of MAO-B. HMC bound to MAO-B with a binding energy of −7.3 kcal/mol, which was greater than its affinity with MAO-A (−6.1 kcal/mol), indicating that HMC is a more potent and selective inhibitor for MAO-B than MAO-A. In addition, HMC showed pharmacological advantages as it has high gastrointestinal absorption, passes through the blood–brain barrier, and is non-toxic. The results in this study suggest that ELF can be an excellent resource in exploring new pharmaceuticals.

## Figures and Tables

**Table 1:** Inhibitory activities of monoamine oxidase-A (MAO-A), MAO-B, acetylcholinesterase (AChE), and butyrylcholinesterase (BChE) by endogenous lichen fungi (ELF) extracts.

ELF No.	Residual Activity at 20 µg/mL (%)			
	MAO-A	MAO-B	AChE	BChE
13	-	19.7 ± 5.67	-	-
22	-	22.2 ± 6.87	-	-
26	-	26.2 ± 1.40	-	26.3 ± 0.59
68	26.2 ± 5.76	-	-	-
71	43.6 ± 2.47	-	-	-
73	-	28.0 ± 1.32	-	-
74	-	-	41.0 ± 3.83	-
110	-	27.0 ± 1.16	-	-
172	-	-	40.8 ± 1.35	-

Extracts were screened by a single assay of each enzyme inhibition, and then effective extracts were additionally analyzed two times. The cutoff of residual activities were 30% for MAO-B and 50% for MAO-A, AChE, and BChE. The results are shown as mean and standard deviation for triplicate experiments. -, not determined.

**Table 2:** DPPH (2,2-diphenyl-1-picrylhydrazyl) antioxidant activity of endogenous lichen fungi extracts.

ELF No.	Inhibition at 100 µg/mL (%)
	DPPH
8	58.5 ± 1.94
84	39.0 ± 0.33
87	84.8 ± 0.50

% inhibition = (absorbance of control—absorbance of reaction mixture)/absorbance of control × 100. The results are expressed as mean and standard deviation by duplicate experiments.

**Table 3:** Inhibitory activities for the enzymes of two isolated compounds.

ELF13	Residual Activity at 2 µg/mL (%)					% Inhibition ^b^
	MAO-A	MAO-B	AChE	BChE	BACE-1 ^a^	DPPH
**C1**	57.1 ± 2.72	89.3 ± 3.68	81.0 ± 2.01	80.6 ± 1.31	93.4 ± 1.02	9.58 ± 0.29
**C2**	55.8 ± 0.91	13.2 ± 0.48	73.0 ± 1.13	83.8 ± 5.89	98.6 ± 2.04	25.11 ± 2.12

The compounds were separated with the second solvent (chloroform:toluene = 1:9, *v*/*v*). Results are expressed as mean and standard deviation from duplicate experiments. ^a^ Residual activity at 10 µg/mL. ^b^ Concentration at 100 µg/mL.

**Table 4:** Inhibitory activities of HMC against the enzymes.

Compounds	IC_50_ (µM)			
	MAO-A	MAO-B	AChE	BChE
HMC	13.97 ± 2.07	3.23 ± 0.3	>40	>40
Lazabemide	-	0.063 ± 0.015	-	-
Pargyline	-	0.028 ± 0.0043	-	-
Toloxatone	1.08 ± 0.025	-	-	-
Clorgyline	0.0035 ± 0.00067	-	-	-
Donepezil	-	-	0.009 ± 0.0019	0.18 ± 0.0038

Results are expressed as mean and standard deviation from duplicate experiments. -, not determined.

**Table 5:** Predicted pharmacokinetic properties of HMC.

Compound	GI Absorption	BBB Permeant	P-gp Substrate	CYP1A2 Inhibitor	CYP2C19 Inhibitor	CYP2C9 Inhibitor	CYP2D6 Inhibitor	CYP3A4 Inhibitor	Log *K_p_* (Skin Permeation)
HMC	High	Yes	No	YES	No	No	No	No	−5.97 cm/s

GI: gastrointestinal; BBB: blood–brain barrier; P-gp: P-glycoprotein; CYP: cytochrome P450.

**Table 6:** Physicochemical parameters and Lipinski violations.

Compound	Mw(g/mol)	cLog *P*	HBD	HBA	TPSA (Å²)	RB	Lipinski Violations
HMC	178.18	1.70	1	3	46.53	No	0

Mw: molecular weight; cLog *P*: consensus Log *P°*_/*w*_; HBD: H-bond donors; HBA: H-bond acceptors; TPSA: topological polar surface area; RB: rotatable bonds.

## Data Availability

Not applicable.

## Supplementary Materials

The following are available online at https://www.mdpi.com/2309-608X/7/2/84/s1, Figure S1: Inhibitory activity of ELF 195 extracts against MAO-A at 20 µg/mL, Figure S2: Inhibitory activity of ELF 195 extracts against MAO-B at 20 µg/mL, Figure S3: Inhibitory activity of ELF 195 extracts against AChE at 50 µg/mL, Figure S4: Inhibitory activity of ELF 195 extracts against BChE at 50 µg/mL, Figure S5: Antioxidant activity of ELF 195 extract using DPPH at 100 µg/mL, Figure S6: ^1^H NMR spectrum of compound C2, Figure S7: ^13^C NMR spectrum of compound C2, Figure S8: HMBC NMR spectrum of compound C2, Figure S9: LC/MS chromatogram of compound C2, Figure S10: Plots of root mean square deviation during 1000 ps MD simulation of MAO-B in complexes with (*R*)- and (*S*)-HMC, Figure S11: Plots of root mean square deviation during 1000 ps MD simulation of MAO-A in complexes with (*R*)- and (*S*)-HMC.
