# Supplementary material for: Selective Inhibition of Human Monoamine Oxidase B by 5-hydroxy-2-methyl-chroman-4-one Isolated from an Endogenous Lichen Fungus Daldinia fissa
Source: J Fungi (Basel). 2021 Jan 26;7(2):84. doi: 10.3390/jof7020084 (PMC7911959; doi:10.3390/jof7020084)
Supplement: Supplementary file 1 [file jof-07-00084-s001.pdf]

Supplementary Information

Selective Inhibition of Human Monoamine oxidase B by 5-Hydroxy-2-Methyl-Chroman-4-One Isolated from an Endogenous Lichen Fungus *Daldinia fissa*

Geum Seok Jeong [1], Myung-Gyun Kang [2], Sang-Ah Han [3], Ji-In Noh [1], Jong Eun Park [1], Sang-Jip Nam [4], Daeui Park [2], Sung-Tae Yee [1] and Hoon Kim [1,*]

Table of Contents

Figure S1. Inhibitory activity of ELF 195 extracts against MAO-A at 20 μg/mL……...S3

Figure S2. Inhibitory activity of ELF 195 extracts against MAO-B at 20 μg/mL…….S4

Figure S3. Inhibitory activity of ELF 195 extracts against AChE at 50 μg/mL…......…S5

Figure S4. Inhibitory activity of ELF 195 extracts against BChE at 50 μg/mL…….…S6

Figure S5. Antioxidant activity of ELF 195 extract using DPPH at 100 μg/mL…......S7

Figure S6. ^{1}H NMR spectrum of compound **C2** in MeOD-d_4…………..…………..….S8

Figure S7. ^{13}C NMR spectrum of compound **C2** in MeOD-d_4………..……….……S9

Figure S8. HMBC NMR spectrum of compound **C2** in MeOD-d_4………..……..….S10

Figure S9. LC/MS chromatogram of compound **C2**……………………......………S11

Figure S10. Plots of root mean square deviation during 1 ns MD simulation of MAO-B in complexes with (*R*)- and (*S*)-HMC. ……………..……………...………S12

Figure S11. Plots of root mean square deviation during 1 ns MD simulation of MAO-A in complexes with (*R*)- and (*S*)-HMC. ………………………..…...………S12

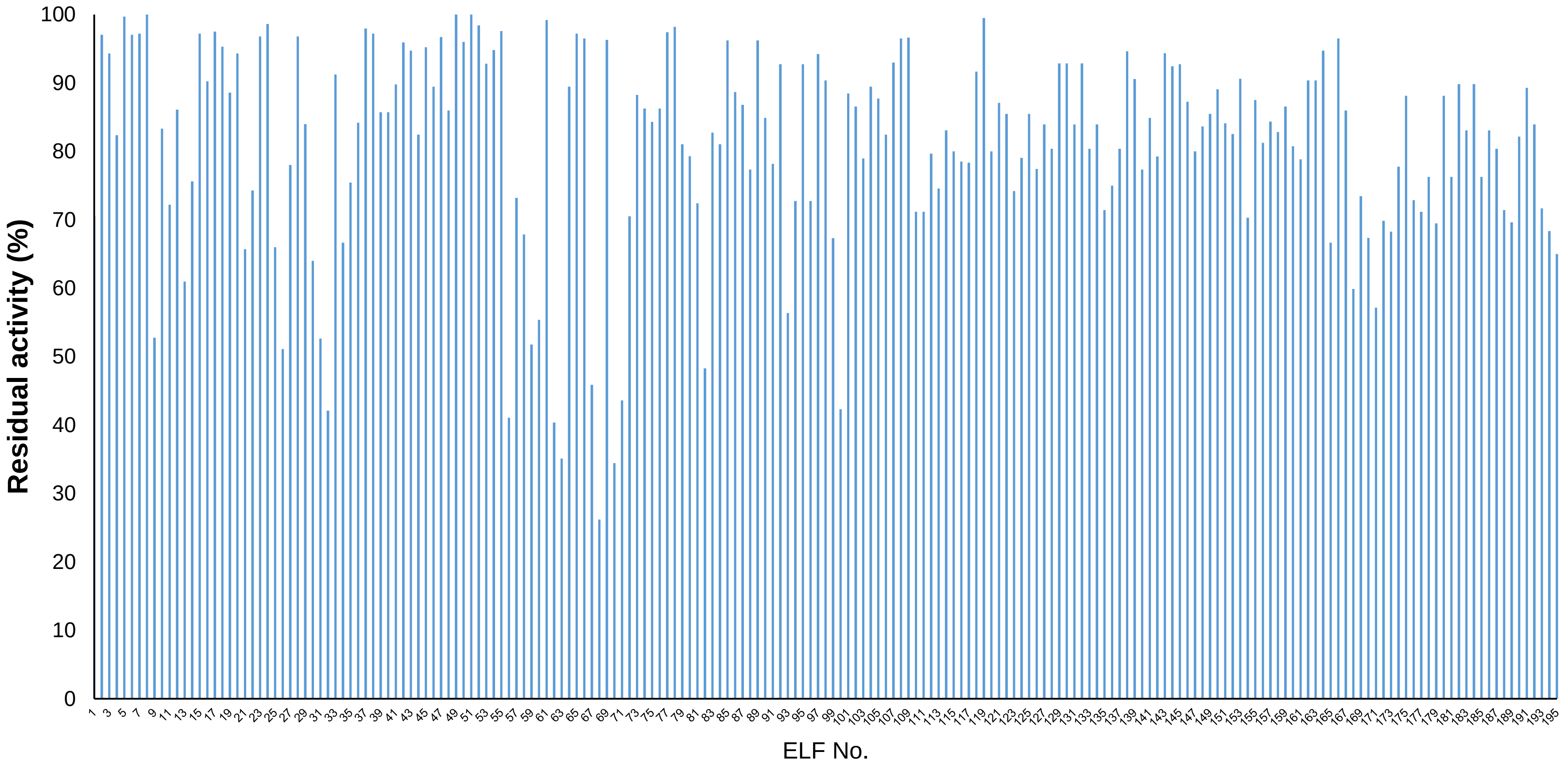

Figure S1. Inhibitory activity of ELF 195 extracts against MAO-A at 20 μg/mL.

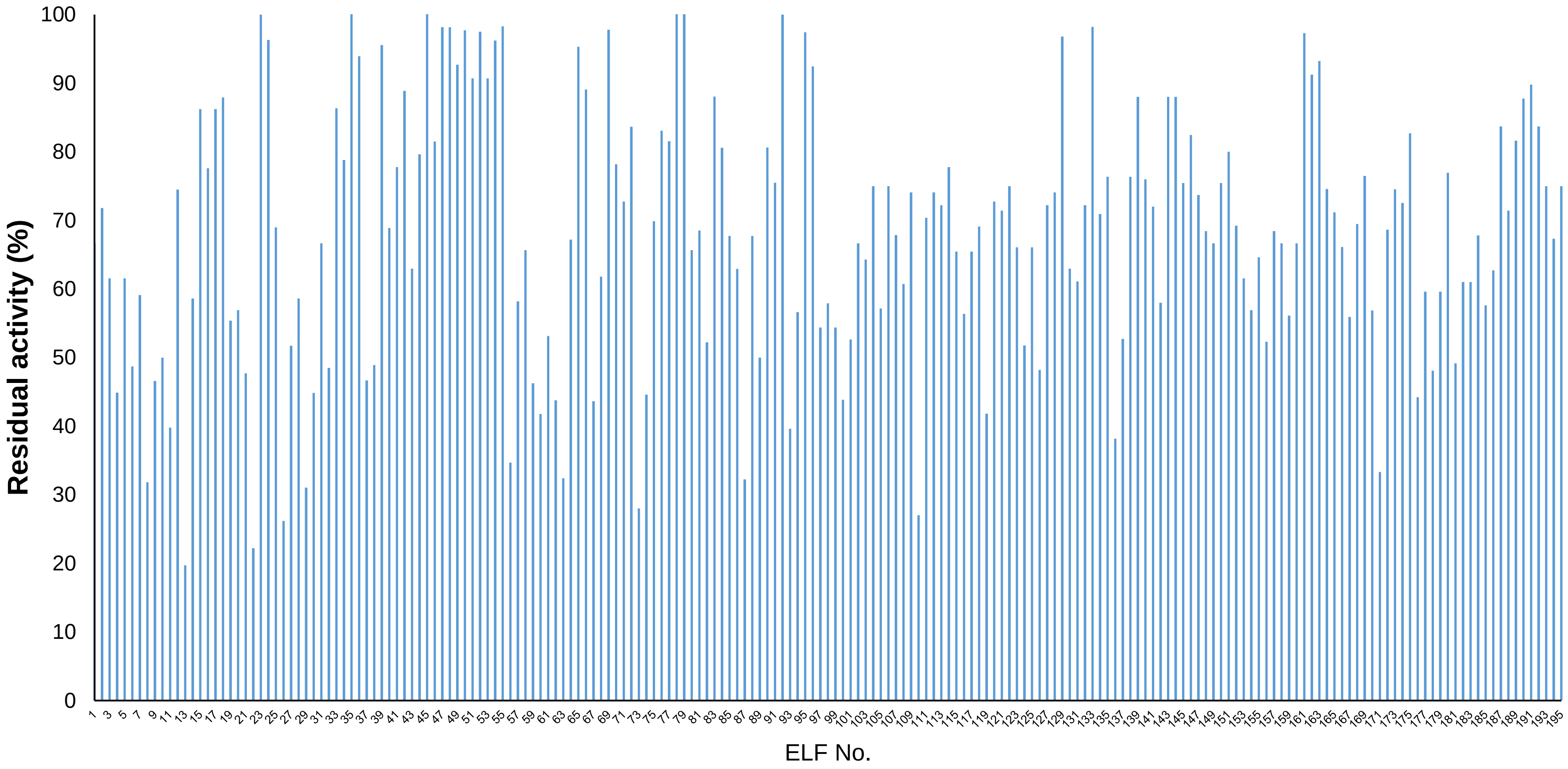

Figure S2. Inhibitory activity of ELF 195 extracts against MAO-B at 20 μg/mL.

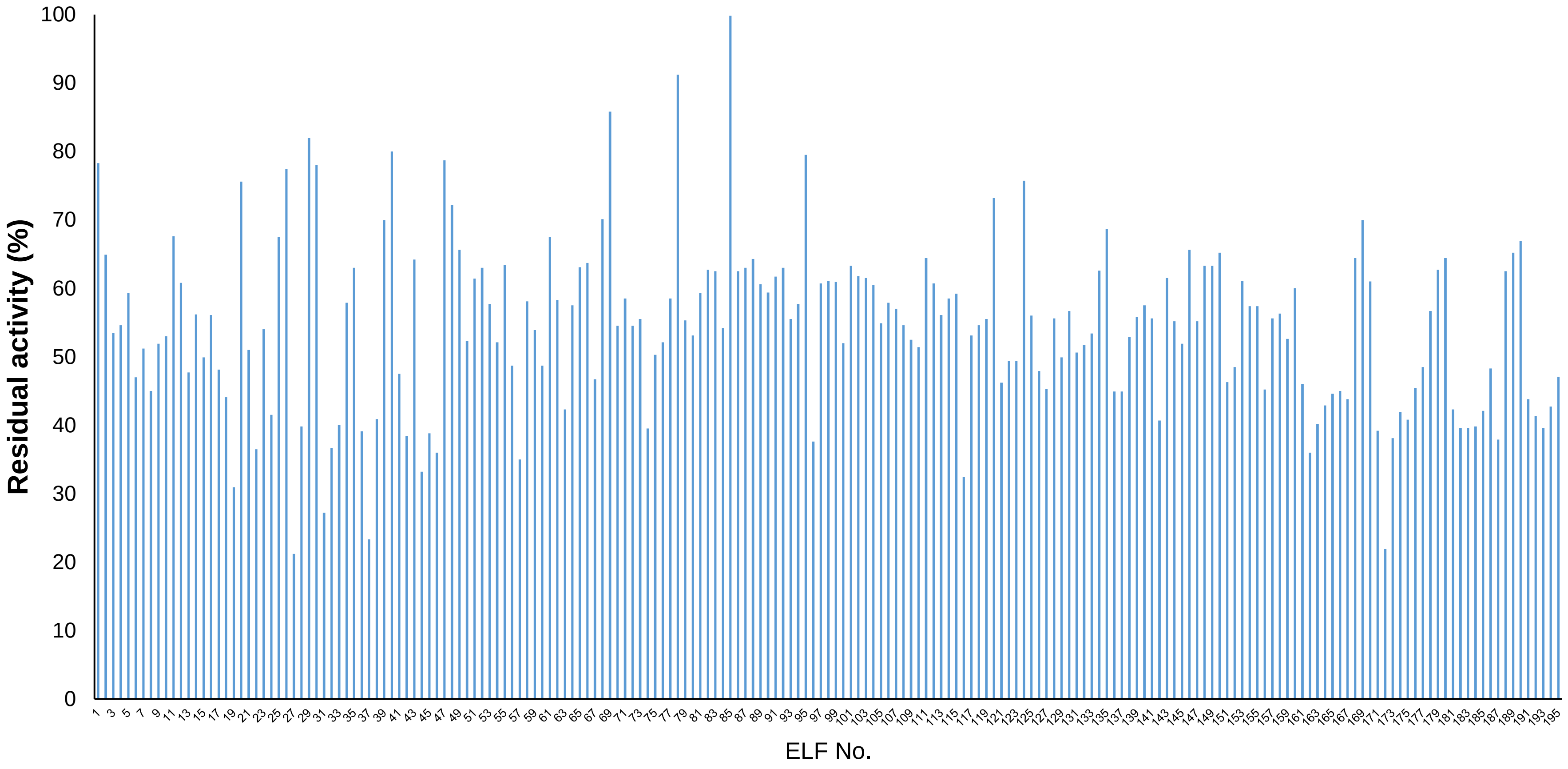

Figure S3. Inhibitory activity of ELF 195 extracts against AChE at 50 μg/mL.

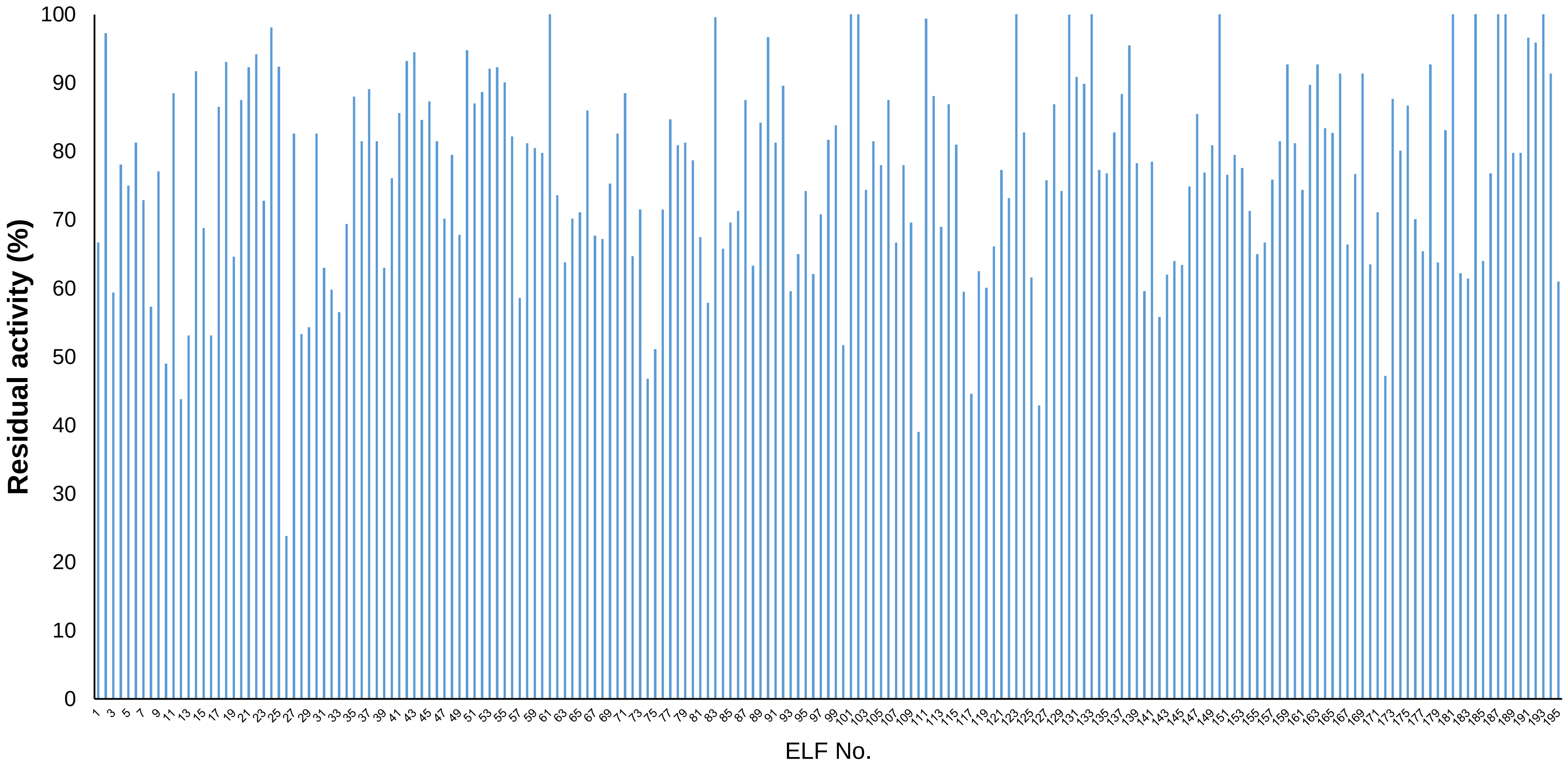

Figure S4. Inhibitory activity of ELF 195 extracts against BChE at 50 μg/mL.

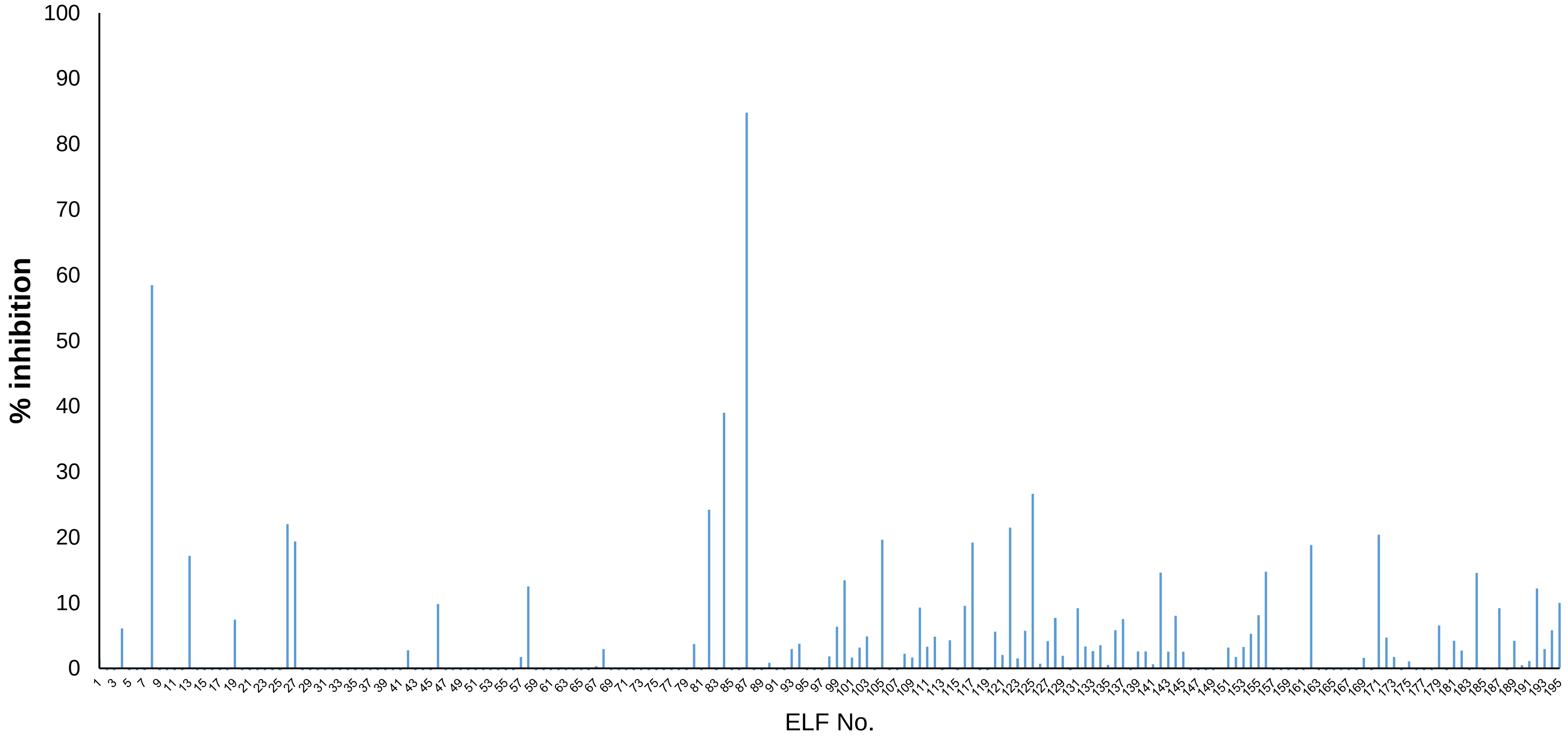

Figure S5. Antioxidant activity of ELF 195 extract using DPPH at 100 μg/mL.

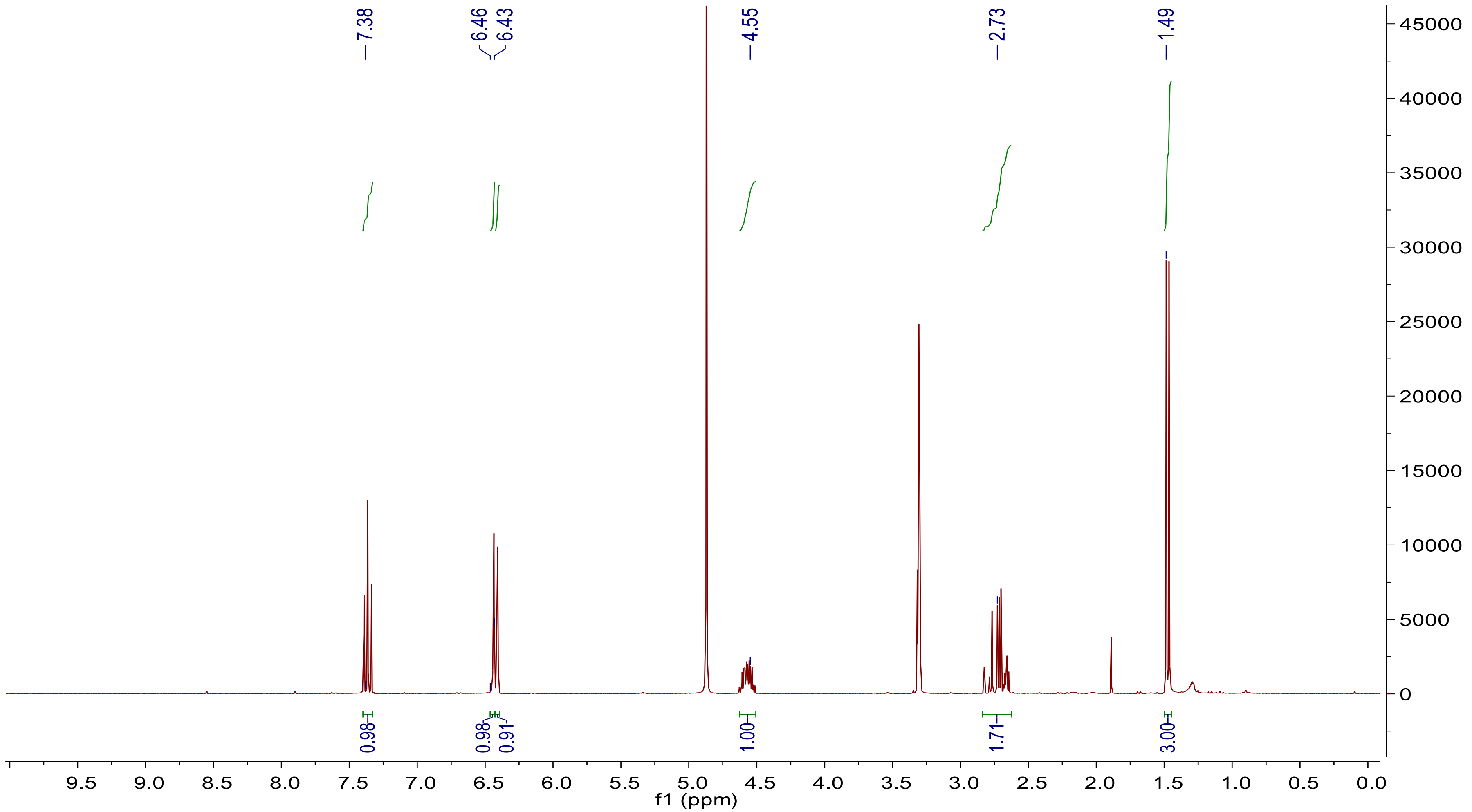

Figure S6. ^{1}H NMR spectrum of compound **C2**.

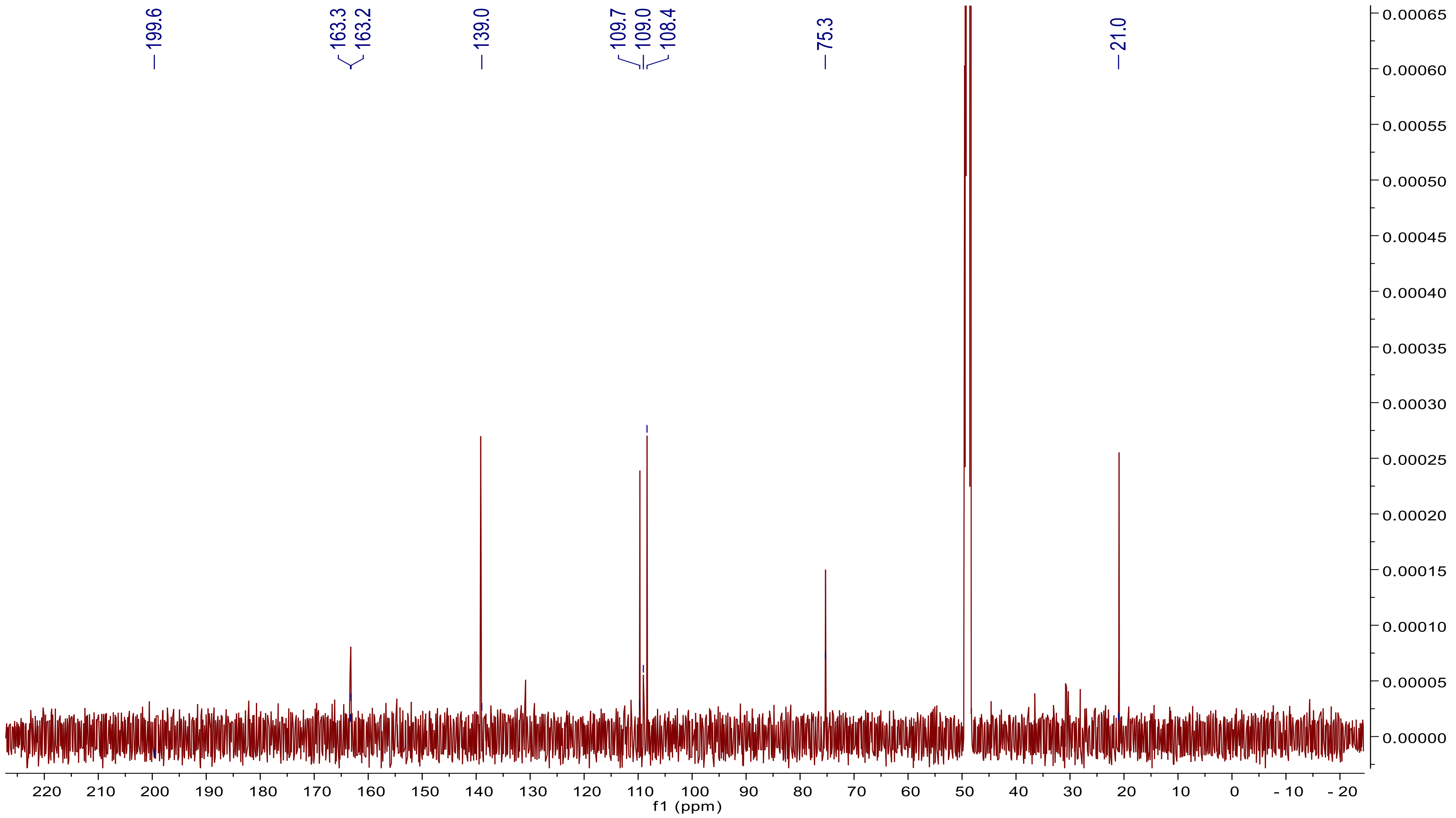

Figure S7. ^{13}C NMR spectrum of compound **C2**.

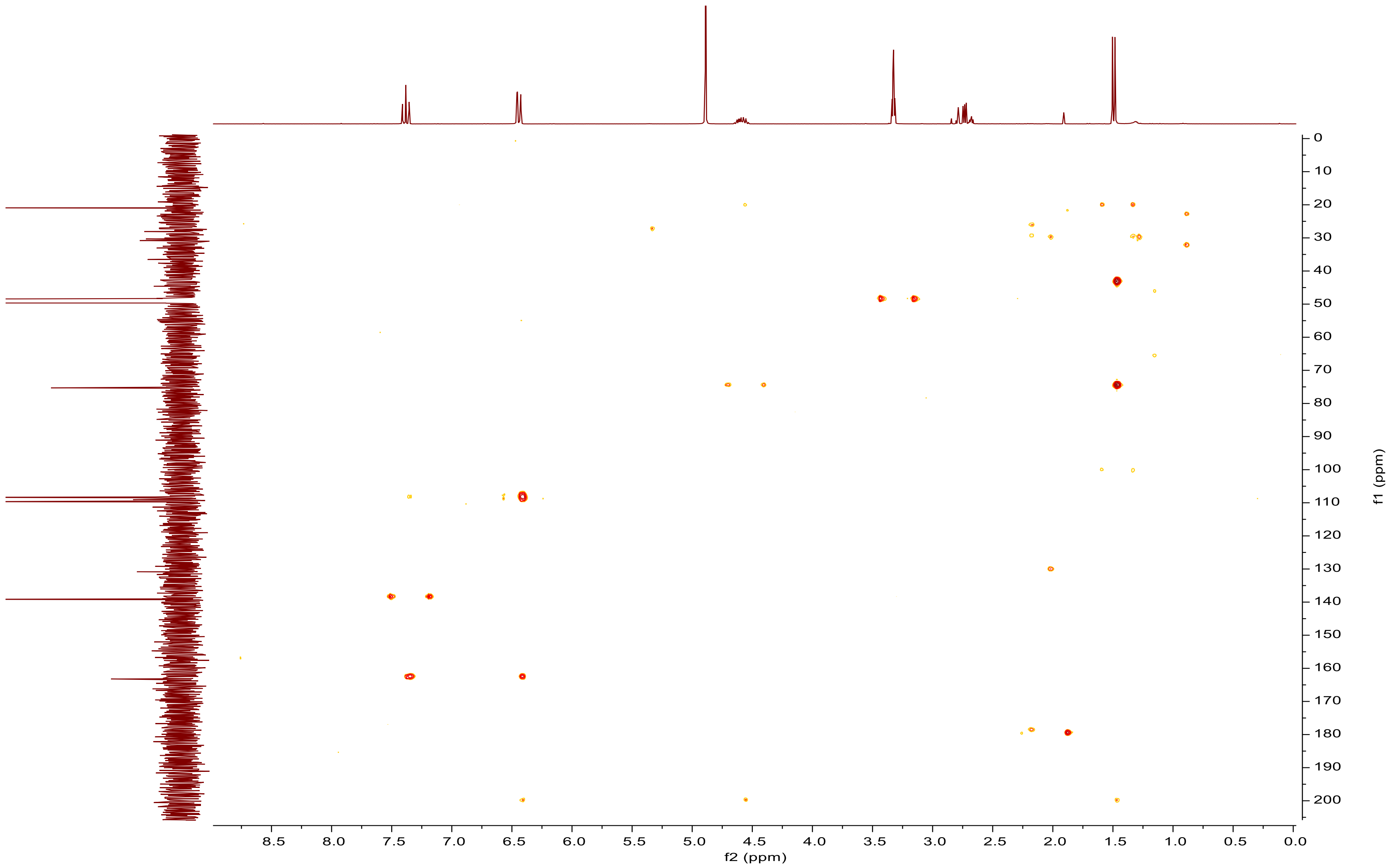

Figure S8. HMBC NMR spectrum of compound **C2**.

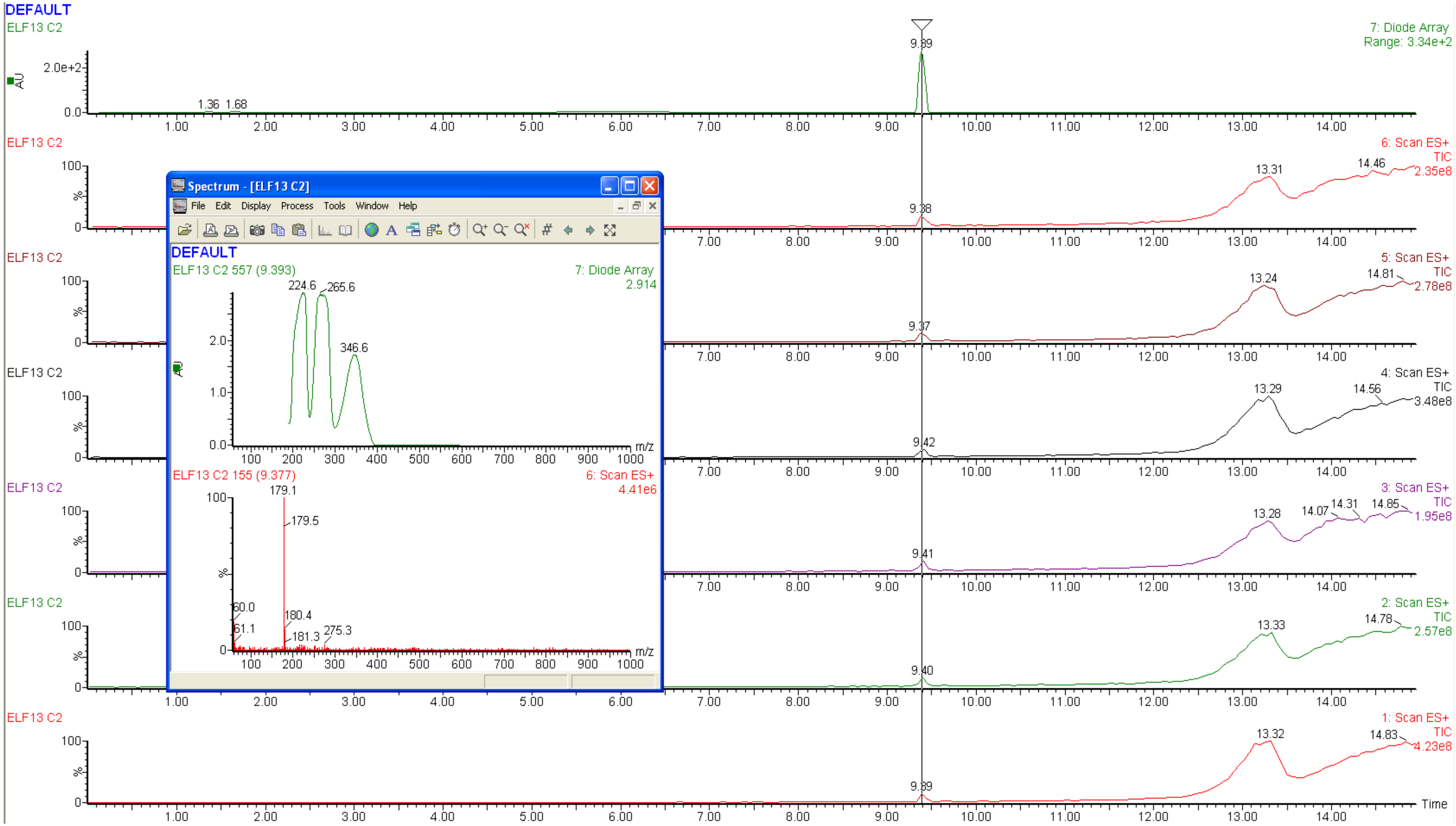

Figure S9. LC/MS chromatogram of compound **C2**.

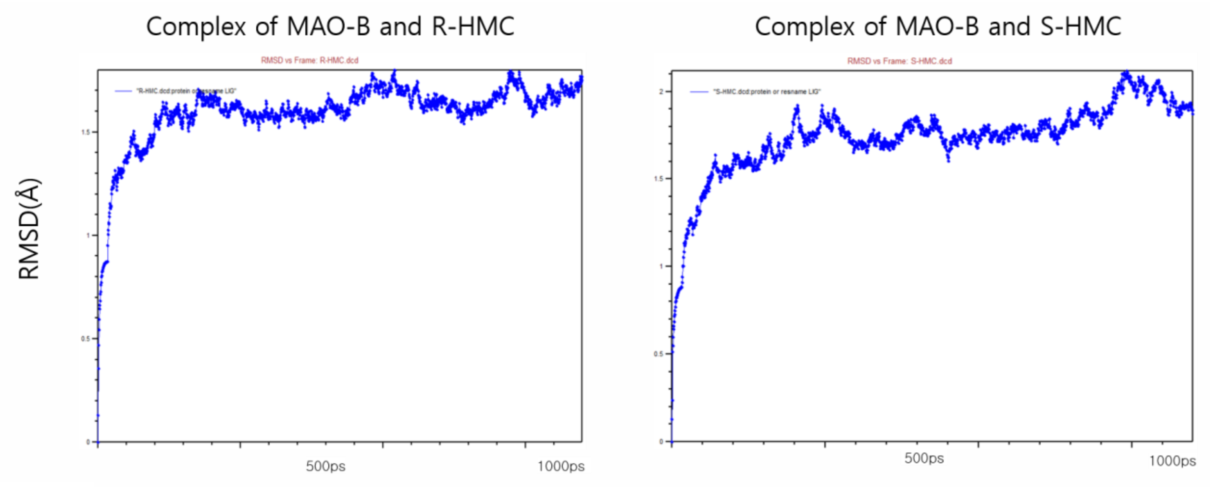

Figure S10. Plots of root mean square deviation during 1000 ps MD simulation of MAO-B in complexes with (*R*)- and (*S*)-HMC.

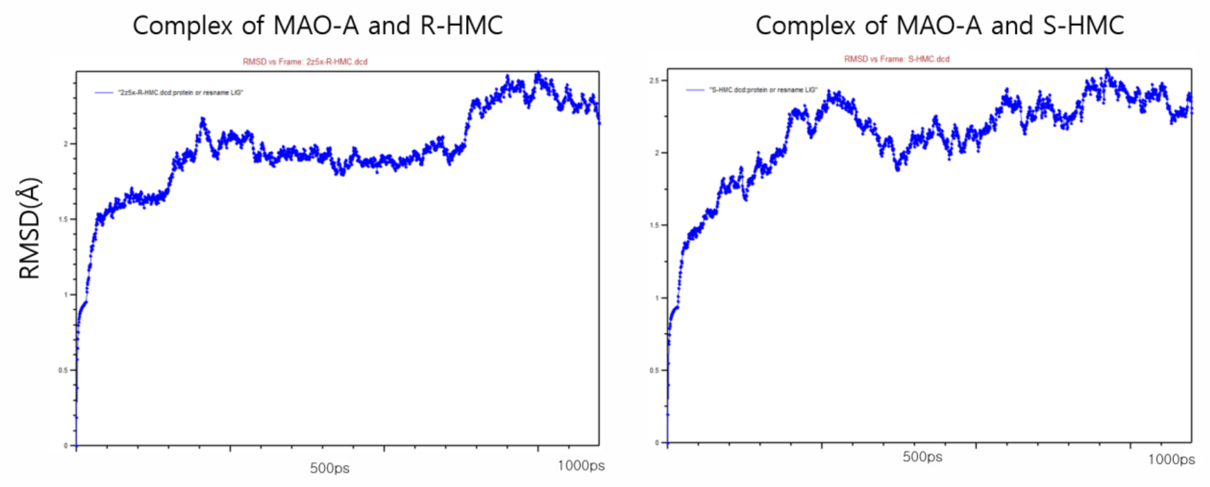

Figure S11. Plots of root mean square deviation during 1000 ps MD simulation of MAO-A in complexes with (*R*)- and (*S*)-HMC.
